# Perspectives of Induction With Chemo and/or Immune Check Point Inhibition in Head and Neck Organ Preservation Treatment

**DOI:** 10.3389/fonc.2019.00191

**Published:** 2019-03-26

**Authors:** Susanne Wiegand, Gunnar Wichmann, Andreas Dietz

**Affiliations:** Department of Otolaryngology, Head and Neck Surgery, University of Leipzig, Leipzig, Germany

**Keywords:** PD-1, PD-L1, neoadjuvant, chemotherapy, immunotherapy

## Abstract

Induction chemotherapy (ICT) is an attractive option for advanced head and neck squamous cell carcinoma (HNSCC) patients which has been prospectively evaluated in the context of a multimodality treatment approach. The theoretical benefit is the ability to suppress distant metastases and shrink the tumor while chemotherapy is better tolerated when given sequentially than concurrently. However, clinical trials have failed to show consistent benefit of ICT over concurrent radio-chemotherapy and due to so far lacking level 1 evidence ICT outside larynx organ preservation remains rather investigational. Immune modulation by inhibition of immune checkpoints is an exciting recent development in HNSCC which has mainly been investigated as second line treatment after progression on platinum-based chemotherapy in patients with recurrent/metastatic HNSCC. Due to the promising results in these trials and even more in the first-line trial KEYNOTE-048 and encouraging first preliminary results of preoperative Anti-PD1-application, the role of neoadjuvant immunotherapy is currently under investigation in HNSCC.

## Induction Chemotherapy

This review gives a general overview of induction chemotherapy (ICT) in squamous cell carcinoma of head and neck (HNSCC). For a more focused review on larynx preservation (LP) see the review of Dietz et al. in this Frontiers special edition.

The idea of ICT followed by radiotherapy (RT) in HNSCC is that chemotherapy is most effective in previously untreated patients and should promote tumor shrinkage, improve local-regional therapy and eliminate micrometastases to reduce the risk of distant metastasis and prolong overall survival (OS). Of course, chemotherapy should be and [as demonstrated by a number of phase-II larynx preservation (LP) trials] is better tolerated reducing acute and late toxicity when given sequentially than concurrently with radiotherapy. A further empirically well described rationale behind ICT is the so-called chemoselection of radiosensitive tumors since chemosensitive tumors also tend to be rather radiosensitive. As there is currently no biomarker predictive for response to chemoradiation, ICT can be used to guide decision-making on further therapy: good responders will receive concurrent chemo-radiation (CRT) and poor responders will receive surgery (1–3). This effect was further explored in LP trials (4–7). In a meta-analysis of ICT as a selection marker for CRT in the head and neck ICT was confirmed to be a predictor for the response to chemoradiation with a pooled median sensitivity of 0.95 (95% CI: 0.72–0.98) and specificity of 0.43 (95% CI: 0.00–0.61). At 2 years, good response to ICT was a statistically significant prognostic marker for OS with a risk ratio of 1.35 (8).

Numerous trials have been performed to investigate the role of ICT for HNSCC in the last decades, but there are no clear guidelines concerning its use in HNSCC outside the recommendations from 2009 (9). Until now, there is still no strong evidence from randomized clinical trials (RCT) that ICT+RT improves outcomes compared to concurrent cisplatin-based CRT for locally advanced HNSCC. LP is currently the only generally accepted setting for whom ICT+RT has a consensus value in HNSCC based on the outcome observed within the landmark Veterans Affairs trial (10). The VA trial by achieving equal OS established ICT with platinum plus 5-FU (PF) followed by RT as an alternative to total laryngectomy (10). The MACH-NC meta-analysis demonstrated that ICT with PF led to an absolute improvement in 5-year OS of 2.4% compared to definitive radiotherapy alone and reduced the rate of distant metastases by 4.3%, but improved locoregional failure rate by only 1% (11). Two RCT showed that ICT with docetaxel (T), cisplatin, and 5-fluorouracil (TPF) is superior to PF regarding locoregional control and OS in patients with HNSCC when given before either radiation alone (TAX323) or CRT with carboplatin (TAX324) (12, 13). However, neither of these trials had a control arm with standard CRT. The superiority of TPF over PF regarding locoregional and distant failure rate was confirmed in a meta-analysis (14). Compared to PF, ICT with TPF results in higher rates of acute toxicity, especially myelosuppression, neutropenia, and neutropenic sepsis. Although several schemes have been tested to reduce the acute toxicity of TPF while maintaining its efficacy, no in this respect successful phase III RCT has been published to date. Currently, docetaxel 75, cisplatin 75, and fluorouracil 750 mg/m^2^ seem to be the most efficacious schedule (12–14). Studies on ICT+RT demonstrated a significant tumor regression and a reduced risk of distant metastases, which was also confirmed in a recent meta-analysis that exhibited a lower propensity for distant metastasis by 11.7% (15). However, an improved OS rate for HNSCC patients treated with ICT+RT compared to standard CRT approaches has not been definitively demonstrated although ICT as early systemic therapy has the advantage of addressing all compartments. However, the beneficial locoregional effect seems to be rather limited although there is better vascularization of tissues prior to any local form of treatment. An exception seems to be laryngeal and hypopharyngeal cancer. The 10-year results of the RTOG 91-11 trial published in 2013 showed a similar efficacy for the endpoint laryngectomy-free survival of PF ICT+RT and concurrent CRT. On the other hand, the authors concluded that locoregional control and LP were significantly improved with concurrent CRT compared to the induction arm (16). Licitra et al. however, demonstrated that the OS (38.8 vs. 27.5%) as well as the survival with larynx after 10 years (28.9 vs. 23.5%) was higher in the induction arm in the RTOG 91-11 trial (17). Forastiere et al. (18) stated in the same issue that the survival differences in the concomitant and induction arm were presented without the overlapping confidence intervals and therefore definitive conclusions regarding survival differences could not be drawn. However, an uncertainty respective to superiority of concurrent CRT over IC+RT exists (17).

In patients with HNSCC outside the larynx ICT was not as successful as in LP RCTs. In patients with advanced oral cancer ICT prior to surgical resection with curative intent failed to improve OS (19). In another meta-analysis of oral cancer patients randomized to either receive ICT or adjuvant to their definitive treatment there was no evidence that ICT improves survival outcomes, but ICT reduced locoregional recurrence (20). Even the use of ICT prior to CRT does not seem to be advisable as meta-analyses and recent trials did not show a survival benefit for patients with locoregionally advanced HNSCC receiving ICT before radiochemotherapy compared to CRT alone (21–23). Taken together, the value of ICT is not finally clarified. Currently, in organ preservation for laryngeal and hypopharyngeal cancers ICT is the most reasonable alternative to concurrent CRT. Attention has been directed toward the identification of patients with an excellent response to ICT who are possible candidates for organ preservation (6, 7). Until now, there is no prospectively approved reliable method for the prediction of response of HNSCC patients to chemotherapy. This causes a possible overtreatment in non-responders by applying ICT without benefit but also potential overtreatment by causing the loss of a functioning larynx due to increased fibrosis or the larynx organ whenever salvage total laryngectomy is required by persistence of disease after administering the full per-protocol treatment. Therefore, the identification of chemotherapy responders before the start of systemic therapy would be a very helpful clinical asset.

## Induction Immunotherapy

Until now, several immunotherapeutic agents that block immune checkpoints, such as those blocking programmed cell death 1 receptor (PD-1) (nivolumab/pembrolizumab/cemiplimab), PD-ligand-1 (PD-L1) (durvalumab/avelumab/atezolizumab), and cytotoxic T lymphocyte-associated protein 4 (CTLA-4) (ipilimumab/tremelimumab), have been investigated and are now approved in different cancer entities. Among these, based on the results from the KEYNOTE-012,−040 and CheckMate-141 trials, nivolumab, and pembrolizumab have already received approval from the FDA and EMA as monotherapy for the treatment of recurrent or metastatic HNSCC in adults progressing on or after platinum-containing chemotherapy (24–26). Pembrolizumab was approved by the EMA only in HNSCC patients whose tumors express PD-L1 with a ≥50% tumor proportional score (TPS). Actually, checkpoint inhibitors look set to revolutionize the treatment of HNSCC and become an integral part of the therapeutic concept. Interim results from the KEYNOTE-048 trial presented at the European Society of Medical Oncology (ESMO) 2018 congress showed that pembrolizumab monotherapy in first-line treatment improved OS by 39% (HR 0.61 [95% CI, 0.45–0.83]; *p* = 0.0007) in patients whose tumors expressed PD-L1 with Combined Positive Score (CPS) ≥20, and by 22% (HR 0.78 [95% CI, 0.64–0.96]; *p* = 0.0086) in patients with CPS≥1 compared to the current standard of care, the EXTREME regimen (PFE; carboplatin/cisplatin, 5-FU, cetuximab + cetuximab maintenance). Moreover, substituting cetuximab by pembrolizumab in the ternary combination with chemotherapy (PF+pembrolizumab) demonstrated improved OS in the first unselected all-comer analysis compared to the EXTREME regimen by 23% (HR 0.77 [95% CI, 0.63–0.93]; *p* = 0.0034), regardless of PD-L1 expression (27).

Immunotherapy by immune checkpoint blockade has a mechanism of action distinct from chemotherapy. Although chemotherapy interacts with the immune system, can promote tumor-targeting immune responses and modulates the composition and functionality of the tumor infiltrate the main action of chemotherapy is that it directly kills tumor cells, whereas the blockage of the PD-1/PD-L1-axis for example always requires an interaction between tumor cells, T-cells, and antigen-presenting cells. Hypothetically, induction immunotherapy may be more effective in HNSCC having a prominent immune phenotype than adjuvant immunotherapy since a higher load of tumor antigens likely exists if the primary tumor is still *in situ* and a larger tumor mass is present (28). In a murine model, neoadjuvant immunotherapy has been significantly more efficacious than adjuvant immunotherapy because of a stronger systemic antitumor T-cell response carried by cytotoxic T lymphocytes (CTL) (20). Inducing a systemic immune response just before surgery could not only activate effector CTL deleting tumor cells expressing tumor-associated antigens (TAA) but also lead to immunologic memory that may protect against tumor recurrence over a long time by eliminating neoplastic cells presenting TAA-derived peptides in their major-histocompatibility-complex class I proteins (HLA-A, B, and C in man) leading to effective immune surveillance (29, 30).

Numerous phase I-III trials investigating PD-1/PD-L1 and/or CTLA-4 inhibitors are being initiated or are already running mainly investigating second line treatment after progression on platinum-based chemotherapy in patients with recurrent or metastatic HNSCC. However, the success of induction immunotherapy in treating other cancers provides rationale for its neoadjuvant use in HNSCC. Until now, interim results of three trials assessing anti-PD-1 treatment in HNSCC were published. Since it will be years before definitive survival outcomes are available, the assessment of pathologic response could provide an early indication of therapeutic efficacy.

CheckMate 358 explores the safety and feasibility of neoadjuvant nivolumab in patients with resectable squamous cell carcinoma of the oral cavity, pharynx, or larynx with T1 or greater primary lesions and N1 or greater nodal disease. Patients receive two doses of nivolumab 240 mg on days 1 and 15 and surgery on day 29 ± 7. The data of 29 patients were published until now. As of database lock, nivolumab-induced preoperative tumor reduction per computed tomography was observed in 11 of 23 (48%) evaluable patients; 3 of them had a tumor reduction ≥40% (largest reduction 75%). Nivolumab was well tolerated, treatment-related adverse events of grade 3–4 occurred in 4 patients (lipase increased; glossodynia); no new safety signals were identified. These adverse events did not result in any protocol-defined surgery delays (31).

The preliminary results of 28 patients enrolled in the NCT02641093 trial, a phase II study of neoadjuvant pembrolizumab and adjuvant combined cisplatin and radiation with pembrolizumab in resected HNSCC, were presented at the ASCO annual meeting 2018. Clinically high risk (T3/T4 category and/or ≥2 N+) patients received one dose of pembrolizumab (200 mg) 1–3 weeks before surgical resection. Adjuvant concurrent pembrolizumab (q3 weeks x 6 doses) and radiation (60-66 Gy) were administered, along with weekly 40 mg/m^2^ cisplatin for patients with high risk pathologic features. Forty seven percent of the patients had a pathological response >10%, 32% a major response >70%, and one of those had a pathological complete response after one dose of pembrolizumab. Increased tumor immune cell infiltration predicted pathological assessed response (32).

Uppaluri et al. presented preliminary data of the first 21 enrolled patients of the NCT02296684 phase II trial exploring neoadjuvant plus post-operative adjuvant pembrolizumab in surgically resectable HPV-negative, stage III/IV HNSCC. All eligible patients received one dose of pembrolizumab (200 mg) prior to surgery and those with high-risk pathologic features received postoperative adjuvant cisplatin and radiation followed by pembrolizumab. Forty three percent of the patients had a pathologic treatment response >10% to neoadjuvant pembrolizumab and 48% of the patients had a clinical-to-pathologic downstaging. A pathologic treatment effect in ≥70% of the resected tumor or lymph node tissue area occurred in 29% of the patients. A significant correlation between baseline PD-L1 expression on tumor cells and pathologic treatment effect in the tumor could be shown. There were no serious study drug-related adverse events or unexpected surgical delays and no locoregional recurrence or distant metastasis in the first 10 patients within more than 1-year follow-up after surgery (33).

A comprehensive list of ongoing trials in HNSCC patients investigating the use of PD-1/PD-L1 inhibition in the neoadjuvant/induction setting can be found in Table 1.

**Table 1:** Ongoing trials in HNSCC patients investigating the use of induction immunotherapy with PD-1/PD-L1 inhibitors.

**Trial**	**Phase**	**Patient population**	**Neoadjuvant treatment**	**Definitive treatment**	**Adjuvant treatment**	**Start date**	**Estimated primary completion date**	**Primary outcome measure**
NCT02641093	II	Resectable, locally advanced HNSCC	Pembolizumab	Surgery	Radiotherapy +pembrolizumab ± cisplatin	Jan 2016	Jul 2019	Treatment-related adverse events; disease-free survival
NCT02919683	II	Resectable oral cancer ≥T2	Nivolumab vs. nivolumab+ ipilimumab	Surgery	Standard	Nov 2016	Apr 2020	Response rate
NCT02296684	II	Resectable, locally advanced HNSCC stage III+IV	Pembrolizumab	Surgery	Radiotherapy ± pembrolizumab ± cisplatin	Mar 2015	Mar 2020	Locoregional recurrences rate; distant failure rate; rate of major pathologic treatment effect
NCT03021993	II	Resectable locally advanced oral cancer	Nivolumab	Surgery	Standard	May 2017	Apr 2019	Pathologic response rate
NCT02488759	I/II	Resectable SCC of the oral cavity, larynx, pharynx >T1 >N1	Nivolumab vs. nivolumab + ipilimumab vs. nivolumab + relatilimumab vs. nivolumab + daratunumab	Surgery	Standard	Oct 2015	May 2019	Drug related adverse events and serious adverse events; rate of surgery delay; objective response rate
NCT02827838	Ib	Resectable SCC of the oral cavity or oropharynx	Durvalumab	Surgery	Standard	Jan 2017	Jul 2019	Local and systemic immune response; effects on immune regulatory mechanisms
NCT03174275	II	Resectable HNSCC stage III-IV	Carboplatin + paclitaxel +durvalumab	Surgery	Durvalumab vs. durvalumab + CRT	Dec 17	Sep 2021	Pathologic complete response rate
NCT03700905	III	Resectable stage III-IVB HNSCC	Nivolumab vs. nivolumab + ipilimumab	Surgery	RT vs. CRT vs. (C)RT + nivolumab vs. (C)RT with nivolumab +ipilimumab	Aug 2018	May 2024	Disease-free survival
NCT03737968	II	Resectable stage II-IV HNSCC	Durvalumab vs. durvalumab + tremelimumab	Surgery	(C)RT + durvalumab maintenance	Jan 2019	Dec 2022	Locoregional relapse rate; distant metastatic rate
NCT03325465	II	HNSCC T1N1-N2b, T2-T4n0-N2b	Pembrolizumab + epacadostat	Surgery	Pembrolizumab + epacadostat ± (C)RT	Nov 2018	Jun 2020	Treatment response rate; rate of complete response
NCT02997332	I	Resectable HNSCC	Durvalumab + docetaxel + cisplatin + 5-flourouracil	Surgery	Standard	Mar 2017	Dec 2019	Recommended phase II dose; number of dose limiting toxicity
NCT02882308	II	Resectable HNSCC	Olarparib vs. cisplatin and olaparib vs. olaparib + durvalumab	Surgery	Standard	Oct 2016	Apr 2019	Change in the tumor Ki-67
NCT03708224	II	Resectable HNSCC stage III-IV	Atezolizumab vs. atezolizumab +emactuzumab	Surgery	Standard	Nov 2018	Mar 2022	Proportion of subjects with a ≥40% increase in the CD3 counts R0 resection rate
NCT03003637	I/II	T3-4N0-3M0 HNSCC	Nivolumab vs. nivolumab + ipilimumab	Surgery	Standard	Feb 2017	Aug 2019	Delay in surgery; pathological response; potential impact of local tumor hypoxia on tumor T-cell abundance and capacity
NCT03700905	III	Resectable HNSCC stage III-IVb	Nivolumab	Surgery	(C)RT + nivolumab vs. (C)RT+ nivolumab +ipilimumab	Aug 2018	May 2024	Disease-free survival
NCT03721757	II	T1-4 N1-3 or any T3-4 N0 oral cavity SCC	Nivolumab	Surgery	(C)RT + nivolumab	Nov 2018	May 2021	Disease-free survival; recruitment rate
NCT03575234	I	Stage III-IVA oral cavity cancer or HPV+ oropharyngeal cancer	Nivolumab, cyclophosphamide, IRX-2	Surgery	Standard	Jul 2019	Jan 2025	Incidence of adverse events; changes in tumor size
NCT03247712	I/II	Resectable HNSCC	Nivolumab + radiation	Surgery	Standard	Jan 18	Dec 2025	Patients with an unplanned delay to surgery
NCT03342911	II	Resectable HNSCC	Nivolumab, carboplatin, paclitaxel	Surgery	Standard	Nov 2017	Jun 2020	Pathologic response rate
NCT03107182	II	Locoregionally advanced HPV+ oropharynx Cancer	Carboplatin, paclitaxel, nivolumab	Surgery vs. RT vs. CRT	Nivolumab	Jun 2017	Jun 2020	Tumor shrinkage
NCT03114280	I/II	Untreated, unresectable, locally advanced HNSCC, stage III or IV	Docetaxel + cisplatin + 5-fluorouracil + pembrolizumab	CRT with carboplatin	–	Nov 2018	Nov 2020	Progression-free survival
NCT02777385	II	Intermediate or high-risk, previously untreated, locally advanced HNSCC	Pembrolizumab	CRT with cisplatin +pembro-lizumab	–	Jun 2016	Apr 2019	1-year progression-free survival;1-year failure rate; acute toxicity rate

Many other immune checkpoint inhibitors are on the horizon and are currently evaluated in the neoadjuvant setting in HNSCC. Interim results of a phase I trial (NCT02540928) investigating AMG 319, an orally bioavailable small molecule inhibitor of the delta isoform of phosphoinositide-3 kinases (PI3K) with potential immunomodulating and antineoplastic activities, were presented at ASCO 2018. The authors concluded that the percentage inhibition that occurred in HNSCC is similar in magnitude to that of patients with advanced B-cell malignancies, and thus supports target inhibition (34). A further example is a phase Ib clinical trial (NCT02274155) investigating an agonistic antibody to OX40 (MEDI6469) prior to surgery of HNSCC. First results demonstrated preoperative MEDI6469 administration to be safe, resulting in increased activation and proliferation of T-cells within the tumor which peaked 2 weeks following infusion (35).

Although these data demonstrate that induction (neoadjuvant) immunotherapy is safe, it must be considered that severe immune-mediated toxicities potentially could occur. On the other hand, quality-of-life appears to be substantially better in patients receiving immunotherapy compared to chemotherapy. In the CheckMate-141 and KEYNOTE-40 trials, nivolumab, and pembrolizumab showed improved quality-of-life outcomes compared to single-agent chemotherapy of investigator's choice in patients with platinum-refractory recurrent or metastatic HNSCC (36, 37).

Currently it is not clear whether OS of HNSCC patients receiving induction immunotherapy will be improved. Moreover, it is uncertain whether recurrent tumors will retain responsiveness to PD-1/PD-L1 antibodies. However, an advantage of the neoadjuvant setting is the chance to study the *in vivo* effect of the blockage of the PD-1/PD-L1-axis on the microenvironment in the primary tumor and lymph node metastasis as well as peripheral blood and to gain information about potential biomarkers. The majority of patients with recurrent/metastatic disease in the CheckMate-141 and KEYNOTE-040 trials did not benefit from immunotherapy despite the notable survival benefit obtained with PD-1 antibodies (24, 26). Since immunotherapy is costly and there are possible side effects it is imperative to identify valid predictors of response. Up to now, the detection of PD-L1 is the most common biomarker to optimize patient selection. However, the PD-L1 results differ between different assays as there is no single laboratory test used and there are a variety of cutoffs. In addition, responses occurred in PD-L1-positive and PD-L1-negative tumors which means that some patients lacking PD-L1 expression may still respond to checkpoint inhibitors, whereas patients showing PD-L1 expression do not all benefit from PD-1/PD-L1 antibodies (38). Moreover, the degree of intratumoral heterogeneity in PD-L1 expression is high, therefore estimating the PD-L1 expression status from a biopsy sample may not be that reliable as estimating total tumor load by staining multiple slides for assessing PD-L1 expression status (39). For these reasons the detection of PD-L1 expression alone will not be enough to analyze the responding patient population; to maximize the therapeutic benefit establishment of predictive biomarkers will be important. Gut microbiota also modulates response to anti-PD-1/PD-L1 immunotherapy and bacteria that hamper the treatment have been identified (40). Analysis of the microbiome of patients responding to checkpoint inhibitors could provide information which HNSCC patients are likely to respond to checkpoint inhibition and also how maintaining or modulating the microbiome may render non-responding cancer patients to responders. Moreover, and as suggested by data from our group (41, 42) and others (43), homozygosity in MHC class I antigens represents a barrier to effective immune checkpoint blockade therapy. In melanoma, carrying particular HLA-B antigens (B44) was predictive for increased OS in patients treated with PD-1/PD-L1 and/or CTLA4 blockade and further increased in patients with high tumor mutational burden (TMB; >10 mutations per megabase) (43).

## Combination of Induction chemo- and Immunotherapy

Preclinical studies have shown that chemo- and radiotherapy modify the immune response against tumors (44). In different tumor entities the induction of PD-L1 expression in tumor cells by chemotherapy was demonstrated, thereby sensitizing tumor cells to the subsequent effect of immunotherapy (45–47). Therefore, combinations of immune checkpoint inhibitors and chemotherapy may be successful. First promising results of neoadjuvant combination of immuno- and chemotherapy were reported in non-small cell lung cancer (NSCLC). In a phase II, single-arm, multicenter study (NCT03081689) patients with resectable stage IIIA N2 NSCLC were treated with neoadjuvant paclitaxel, carboplatin and nivolumab. The overall response rate was 84.6 and 69.2% of the patients achieved complete pathologic response (48). Based on this, in the near future there will be a need for clinical trials and translational studies of neoadjuvant combined chemotherapy and immunotherapy in HNSCC. The detection of predictive biomarkers will be important to guide the treatment.

## Conclusion

Actually, TPF is the gold standard for ICT in HNSCC, especially in LP. However, RCT have failed to show consistent benefit of ICT+RT over CRT. Induction immunotherapy is an attractive therapeutic option since the tumor mass of the primary may serve as a rich antigen source for activation of tumor-specific CTL and a systemic immune surveillance (30). Currently, clinical trials investigating immune checkpoint inhibitors in the neoadjuvant setting are underway for patients with HNSCC. The detection of specific biomarkers that predict treatment response will be a prospective challenge to enable selective use of immune therapy in expected responders. The combination of chemo- and immunotherapy will provide additional benefits as demonstrated in NSCLC. In the future, drug selection will probably depend on molecular subtypes and newly discovered biomarkers.

## Author Contributions

All authors listed have made a substantial, direct and intellectual contribution to the work, and approved it for publication.

### Conflict of Interest Statement

The authors declare that the research was conducted in the absence of any commercial or financial relationships that could be construed as a potential conflict of interest.
